# Hahnemann and Public Confidence

**Published:** 1918-04-20

**Authors:** J. Carlton Still

**Affiliations:** Chairman of the Liverpool Hahnemann Hospital. Hope Street, Liverpool


*To the Editor of* THE HOSPITAL.

### "Hahnemann and Public Confidence."

*To the Editor of* THE HOSPITAL.

SIR,—I had hoped that, notwithstanding what has already appeared in the press, I should not have to write anything regarding the Liverpool Saturday and Sunday Fund which might cause the impression that we have a controversy with one of our principal donors; but as you think the position should be made clear I will try quite dispassionately to give you the facts.

According to our printed accounts for the year 1916 there was a balance, being excess of income over expenditure, of £807 7s., which was arrived at after including a donation of £300 in our receipts which in the ordinary course would have been placed in our legacy account, and the normal excess was therefore £505 7s., and we did not carry forward "a credit balance of £805 after investing £300."

This was the first credit balance of ordinary income over ordinary expenditure since the opening of the hospital in 1887.

The result of this unusual experience was that on August 2, 1917, the secretaries of the Fund wrote to us enclosing £50, with the expression of their regret, but stating that the claims of other institutions were so great, and their finances so deplorable, that they felt it incumbent on them to take this course, as on the year's returns supplied to them we showed a substantial credit balance, and they added that they would be prepared to carefully consider our case this year.

This being so, and as the distribution for 1916 had been made, we decided to wait till we saw how this loss affected our position at the end of the year 1917.

Our annual meeting was held on February 21, and the report of the committee was presented. It said that our financial statement showed a loss of £400 12s. 10d. on the year, and that this has arisen through the decrease of the customary grant of the Hospital Saturday and Sunday Fund, the material increase in the cost of maintenance, provisions, etc., and an exceptional loss.

We tender our thanks to the Fund for the gift of £50, we say that the loss of £487, the difference between the £50 and the amount received in 1916, has been a serious one, and we express our regret.

As the letter from the secretaries before referred to offered a careful consideration of our case this year, we shall in due course lay our appeal for the share of the 1918 collection before the committee, and have no reason to doubt that it will receive sympathetic consideration.

In the letter addressed to the Liverpool papers the Fund secretaries say that "two other excellent institutions received similar drastic treatment," and I think they must have realised that this "drastic treatment" might be commented on by both subscribers to the Fund and supporters of the hospital, but we cannot be held responsible for individual criticism, and so far as the official report of the hospital is concerned the committee properly confined itself to a statement of facts, and the Fund can therefore have no ground of complaint against it.

The last paragraph in your article—namely, that "hospitals named after Hahnemann are apt to suppose that they are liable to preferentially less generous treatment than others" does not apply in the case of the Liverpool Hahnemann Hospital. It would be a most unwarrantable accusation against the committee of the Fund and most ungenerous on our part, in view of the annual grant which we have received ever since the opening of the hospital, and I do not believe that our supporters in Liverpool hold that we have received "preferentially less generous treatment" on the ground you name.—Yours faithfully,

J. CARLTON STILL,
Chairman of the Liverpool Hahnemann Hospital.

Hope Street, Liverpool, April 6, 1918.

